# The Protective Role of Ozone Therapy in Kidney Disease: A Review

**DOI:** 10.3390/life13030752

**Published:** 2023-03-10

**Authors:** Luis Fernando Delgadillo-Valero, Estefani Yaquelin Hernández-Cruz, José Pedraza-Chaverri

**Affiliations:** 1 Faculty of Medicine, National Autonomous University of Mexico, Mexico City 04360, Mexico; 2 Laboratory F-315, Department of Biology, Faculty of Chemistry, National Autonomous University of Mexico, Mexico City 04510, Mexico; 3 Postgraduate in Biological Sciences, National Autonomous University of Mexico, Ciudad Universitaria, Mexico City 04510, Mexico

**Keywords:** ozone, ozone therapy, kidney disease, oxidative stress, antioxidants defense, inflammation

## Abstract

Ozone (O_3_) is a reactive oxygen species (ROS) that can interact with cellular components and cause oxidative stress. Following said logic, if O_3_ induces such a stressful milieu, how does it exert antioxidant functions? This is mediated by controlled toxicity produced by low concentrations of O_3_, which enhance the cell’s suppliance of antioxidant properties without causing any further damage. Therapeutic concentrations vary extensively, although 50 µg/mL is commonly used in experimental and clinical procedures, given that augmented concentrations might work as germicides or cause endogenous damage. O_3_ therapy has been shown to be effective when applied before or after traumatic renal procedures, whether caused by ischemia, xenobiotics, chronic damage, or other models. In this review, we focus on discussing the role of O_3_ therapy in different models of kidney damage associated with fibrosis, apoptosis, oxidative stress, and inflammation. We integrate and report knowledge about O_3_ in renal therapy, debunking skepticism towards unconventional medicine, explaining its proven therapeutic properties, and thus providing background for its use in further research as well as in clinical settings.

## 1. Introduction

Kidneys are organs of great biological importance. They receive approximately 25% of the cardiac output, and their main functions are filtrating blood, keeping a homeostatic plasma volume, and regulating vascular pH and ion concentration in circulation, amongst many others. They achieve this by reabsorbing necessary biological metabolites and depurating toxic or unhelpful ones through a complex tubular system formed by nephrons [1]. Kidney diseases are described as a group of multicausal syndromes and impaired diagnostic markers (oliguria, albuminuria, or proteinuria, rise of creatinine excretion, or diminished filtration rate, all of which measure renal function) that can be categorized according to their evolution time in acute kidney injury (AKI), before 7 days; acute kidney disease (AKD), up to 3 months; and chronic kidney disease (CKD), after 3 months. They are highly prevalent and represent a global health issue. For instance, AKI is experienced by 1 in 5 hospitalized patients worldwide [2] and has a high mortality rate of approximately 21%. However, more severe stages, such as patients needing kidney replacement therapy, are associated with higher mortality (46%) and unaffordable costs. In 2012, 864,226 deaths were attributable for CKD and predictions suggest mortality will increase, even when CKD is currently the fourteenth cause of death worldwide, with 12 deaths per 100,000 individuals [3].

Conventional therapeutics as the disease progresses are often targeted only towards reducing risk factors, such as diabetes, hypertension, and dyslipidaemia, which might aggravate the disease and favor its progression by causing glomerulosclerosis, endothelial damage, salt retention, volume overload, and tubular atrophy [3,4]. Such treatments are also intended to compensate diminished renal function. For example, recommendations from the National Institute of Health include diuretics and renin- angiotensin aldosterone system (RAAS) inhibitors, which are not only useful as treatment for hypertension, but might also compensate for lost electrolyte excretion and diminish albuminuria, respectively [5]. Reducing blood pressure is important given the high prevalence of this disease, which is present in about half of the American population [6] and is one of the main causes of CKD along with diabetes, furtherly discussed in the text. Other international treatment guidelines, e.g., the Mexican Clinical Practice Guide, suggest treatment with SGLT-2 inhibitors to reduce CKD progression in diabetic patients, as well as the use of statins to reduce cardiovascular events [7]. Chronic and end stage renal conditions often need kidney replacement therapy, which includes renal transplant, hemodialysis, and peritoneal dialysis treatment [8]. The survival rate in dialyzed patients is approximately 55% after five years, less than expected for the general population [9]. Besides reported oxidative damage observed in CKD and dialyzed patients [10], the Kidney Disease Improving Global Outcomes (KDIGO) 2012 Clinical Practice Guideline for the Evaluation and Management of CKD does not include any antioxidant treatment in its recommendations [11].

Medical costs associated only to free for service Medicare beneficiaries with CKD can sum up to 49 billion dollars each year [12], even when prevention of kidney disease is promoted with strategies, such as weight control, patient education, diet, exercise, and identifying additional risk factors, e.g., dehydration, anemia, cancer, hypovolaemia, hypoxia, use of nephrotoxic drugs, heart failure, sepsis, hepatorenal syndrome, amongst others [13]. Therefore, finding alternative therapies for patients with kidney diseases, including CKD, AKI, or those needing a transplant, is vital. Ozone (O_3_) is a molecule consisting of three unstably bound oxygen atoms that has been used as a novel therapy in many diseases. It is formed naturally when a di-oxygen molecule (O_2_) binds to atomic oxygen, formed by ultraviolet (UV) radiation. Moreover, it is artificially created, using O_3_ concentrators, which work through the electrical discharge of gas, creating an airtight barrier that prevents O_2_ from passing through, forcing it to dissociate and form O_3_ instead [14]. O_3_ is considered a reactive oxygen species (ROS). In other words, it is more reactive than O_2_. However, since it does not have unpaired electrons, it is considered a non-radical one that can react with biological cell structures, such as lipids, proteins, and nucleic acids. O_3_ can also promote reactions that form reactive species, such as hydroxyl radicals; such toxic characteristics have also been described as possible antimicrobial mechanisms [15].

Considering the above factors, the purpose of this review is to integrate and present knowledge about O_3_ in renal therapy, debunking skepticism towards non-conventional medicine, explaining its proven therapeutic properties, and thus providing background for its use in further research as in clinical settings.

## 2. O₃ Mechanism of Action

To understand O_3_ mechanism of action and chemical properties, O_3_ must be identified as a ROS, a molecule with a great oxidizing capacity. ROS are classified as non-radicals and free radicals (with one or more uncoupled electrons). However, O_3_ is among the non-radicals [16]. O_3_ can react by itself with biological structures, such as amino acids (especially aromatic), enzymes, deoxyribonucleic acid (DNA), as well as membrane glycoproteins and lipids. However, due to its instability in the gaseous phase, water solution, or in combination with other extracellular substances, it can dissolve in a chain reaction to form a variety of free radicals, such as hydroxyl radical (^•^OH) and superoxide radicals (O_2_^•−^), which acquire missing electrons by oxidizing biological components. Eventually, when the breakdown of ozone is inhibited, the chain reaction ends. This is because organic and inorganic substances, such as stable carbonate ions (CO_3_^2−^) and bicarbonate (HCO_3_), react with OH radicals to form secondary radicals that do not generate superoxide radicals. Another example of termination is the reaction of two free radicals (^•^OH + HO_2_^•^ → O_2_ + H_2_O) [17].

**Figure 1:**
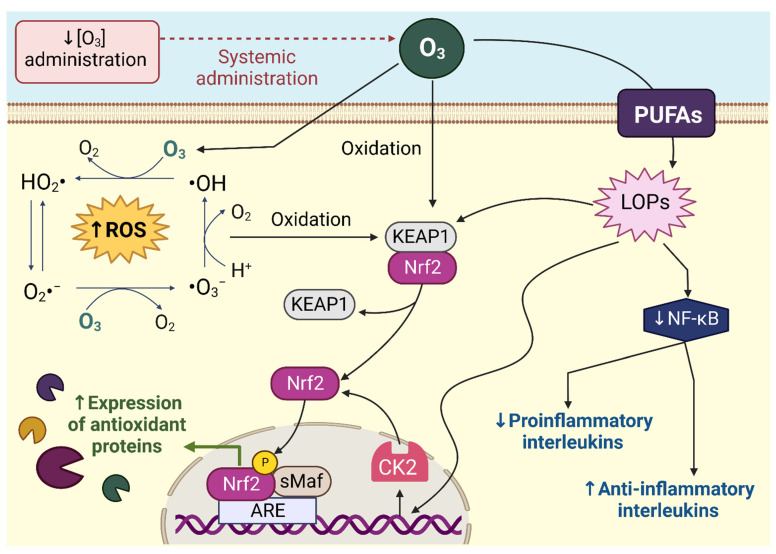
Mechanism of action of the administration of low concentrations of ozone (O₃). O_3_ forms free radicals, such as the hydroxyl radical (•OH) and superoxide radicals (O_2_^•−^), due to different chain reactions. The free radicals formed, and O_3_ oxidized Kelch-like ECH-associated protein 1 (KEAP1), promoting nuclear translocation of nuclear erythroid factor 2 (Nrf2). Nrf2 in the nucleus binds to the antioxidant response element (ARE) and induces the transcription of antioxidant enzymes. Nrf2 nuclear translocation is exacerbated by the action of ozonated lipid products (LOPs) formed by the oxidation of polyunsaturated fatty acids (PUFAs). LOPs act by oxidizing KEAP1 and increasing casein kinase 2 (CK2) synthesis, increasing Nrf2 phosphorylation. In addition, LOPs decreases the expression of nuclear factor kappa B (NF-κB), promising the decrease of proinflammatory cytokines and increasing the concentration of anti-inflammatory cytokines. P: phosphorylation, ROS: reactive oxygen species, sMaf: small musculoaponeurotic fibrosarcoma. Created with Biorender.com, accessed on 10 February 2023.

The ROS created from the interactions of O_3_ and O_3_ itself oxidize the cysteinyl thiols of Kelch-like ECH-associated protein 1 (KEAP1), a protein that keeps nuclear erythroid factor 2 (Nrf2) ubiquitinated and, therefore, destroyed by the proteasome. Such an event leads Nrf2, a transcription factor, to translocate to the nucleus, where it regulates oxidative damage by inducing antioxidant response elements (ARE) [18], including antioxidant enzymes that serve as protective responses that delay, prevent, or remove oxidative damage, via catalytic or scavenger activity of free radicals. Examples of upregulated antioxidant proteins include catalase (CAT), superoxide dismutase (SOD), glutathione peroxidase (GSH-Px), glutathione s-transferase (GSTr), heme oxygenase-1 (HO-1), NADPH quinone oxidoreductase 1 (NQO1), and heat shock protein-70 (HSP70) [19]. O_3_ therapy also works by inducing controlled oxidation of polyunsaturated fatty acids (PUFAs) found in cell membranes. This creates ozonated lipid products (LOPs), which are important signal transducers and regulators of inflammation [20]. LOPs (specifically hydroxy-hydroperoxides) exacerbate Nrf2 nuclear translocation by oxidizing cysteine residues, promoting KEAP1-Nrf2 dissociation and activation of Nrf2-ARE pathways [21,22]. Furthermore, they increase the phosphorylation of Nrf2 by casein kinase 2 (CK2) [21]. In addition, LOPs have a hormetic response since, at low concentrations, they downregulate activation of nuclear factor kappa B (NF-κB) and inflammatory cytokines and increase antioxidant and anti-inflammatory compounds. High concentrations stimulate potential antimicrobial and chemotactic responses by activating phospholipase A2 and phospholipase C, which via intracellular cascades, including that of the arachidonic acid, stimulate the synthesis of NF-κB and, therefore, pro-inflammatory cytokines, e.g., interleukins (IL) 1 and 6 and tumor necrosis factor-alpha (TNF-α), transforming growth factor beta (TGF-β), as well as the cyclooxygenase (COX) [23]. This explains how controlled and nontoxic oxidation by O₃ mediates the upregulation of antioxidant and anti-inflammatory defenses (Figure 1).

O_3_ is applied in situ due to its short life, as it can only be stored for about 40 min at 20 degrees Celsius. Routes of O_3_ dosing vary depending on the goals and location of therapy and include rectal insufflation and autohemotherapy (for systematic effects), as well as topic and infiltrative therapy (for localized effects, such as musculoskeletal and germicide) [19]. On the contrary, administration through the respiratory pathway is contraindicated due to the insufficient antioxidant capacity of this system and the narrowing effects caused by O₃ on the airway [20]. The therapeutic window range is proposed to be between 20 and 80 µg/mL of O_3_ per gram of blood [24].

Therapeutic effects commonly observed in the kidney after the administration of O_3_ administration include amelioration of renal function, measured through plasma clearance of endogenous metabolites, blood urea nitrogen (BUN), and serum creatinine (SCr). Hence, those molecules filtrated and depurated by the kidney [25] at lesser levels after O_3_ treatment infer protection [26,27,28,29,30,31]. In addition, O_3_ therapy has been reported to decrease morphological damage mainly evidenced under photon microscopy, including medullary hemorrhage, tubular necrosis, glomerular damage, collagen deposition, and fibrosis markers, such as α-smooth muscle actin (α-SMA) and TGF [26,27,28,29,30,31,32,33], besides increasing the suppressor of mothers against decapentaplegic-7 (SMAD-7) [33]. Furthermore, O_3_ therapy reduces inflammation, as evidenced by a diminished expression of cytokines, such as IL, TNF-α, monocyte chemoattractant protein-1 (MCP-1), and intercellular adhesion molecule-1 (ICAM-1), as well as the toll Like receptor 4 (TLR 4)-NFkB pathway [26,34]. Another therapeutic effect is the diminishment of lipid peroxidation, which represents the oxidative stress induced by polyunsaturated fatty acids. A useful marker to quantify this is via malondialdehyde (MDA) [35], which diminishes its renal expression when treated with O_3_ [28,29,31,33].

## 3. Damage Models and O_3_ Effect

### 3.1. O_3_ Therapy Protects the Kidney against Ischemic Damage

Ischemic damage in renal tissue occurs when kidneys experiment periods of diminished or restricted blood supply. In contrast, oxidative damage occurs when tissue is re-oxygenated, which might happen during experimental procedures in rats, such as clamping and unclamping renal pedicle, or during renal transplantation [36]. This kind of damage is proposedly produced through xanthine oxidase (XO). This enzyme degrades nucleotides upon cell ischemia. However, after O_2_ reperfusion, XO forms uric acid and high quantities of superoxide radical, which further produces oxidative stress [37]. This explains why treatment with XO inhibitors, such as tungsten [37], allopurinol [38], or even XO knockout models [14], ameliorates ischemia-reperfusion injury (IRI) and oxidative stress after short periods of ischemia. Finding auxiliary treatments for oxidative damage is clinically important since ischemic-producing scenarios are highly prevalent. Just in 2010, for instance, more than 2 million patients received renal transplants [39].

O_3_ therapy has previously been used before IRI (preconditioning) [26,27,28,33,40,41] or after IRI (postconditioning) [29,30,31,32,42] and has been described as a potential treatment (Table 1). O_3_ therapy is demonstrated to act with similar efficacy, but not synergic, to that achieved when IRI preconditioning is made with other protective strategies, such as inducing short, repeated periods of ischemia before the main IRI. This prepares the renal tissue against the IRI via similar controlled mechanisms as that of the O_3_ and is called ischemic (pre)conditioning [43]. Interestingly, when administered after the main IRI, ischemic postconditioning in conjunction with O_3_ therapy upregulate beneficial effects and even diminishes cell death [44]. After transplantation, rats also show a protective effect against the oxidative state when treated with O_3_ [45,46]. Antioxidant enzymes are also upregulated in cultured kidney cells after they were submitted to hypoxia and reoxygenation [47].

Nitric oxide (NO) and NO synthase (endothelial, eNOS, and inducible, iNOS) have been proposed as oxidants that damage renal tubules through highly reactive peroxynitrite [48]. However, NO was found to be a protective mechanism favored by O_3_ therapy against IRI inflammation and vasoconstriction caused by Endothelin-1 [27,28]. In fact, nitrate-derived NO, when applied topically, is an effective therapy against IRI damage [49].

In summary, O_3_ therapy, either before or after IRI, improves kidney damage by decreasing markers of kidney damage, inflammation, and fibrosis. Therefore, it is a good treatment for ischemic injuries such as kidney transplantation, iatrogenic trauma, partial nephrectomy, heart failure, and hypovolemia, among other prevalent clinical conditions that reduce renal blood flow, such as those that produce AKI.

### 3.2. O_3_ Therapy Protects the Kidney against Xenobiotic-Induced Damage

Xenobiotics are exogenous chemicals not synthesized by a certain organism; therefore, they are not essential for its physiological functions and processes. That way, synthetical drugs, metals, and environmental factors, amongst others, are considered as such [50]. In this section, the mechanisms through which some of these xenobiotics cause nephrotoxicity will be discussed; along with the described protective effects of O_3_ therapy against it, looking forward to discovering the usage of new therapeutic alternatives against damaging products we are constantly in contact with (Table 2).

Acetaminophen (APAP), a common anti-inflammatory drug, has been demonstrated to produce severe nephrotoxicity [51]. Proposed mechanisms include APAP’s hepatic degradation and further enzymatic formation of a highly toxic and reactive metabolite, N-acetyl-p-benzoquinone (NAPQI), which glutathione (GSH) normally neutralizes. However, in APAP overdose, NAPQI is formed in major quantities, proving uncontainable by antioxidant enzymes, and therefore producing oxidative damage, especially in proximal tubules [52]. O_3_ therapy has proven to be an effective antioxidant therapy by enhancing antioxidant enzymes and diminishing oxidation [51]. Interestingly, the administration of O_3_ therapy in APAP induced nephrotoxicity, when combined with another antioxidant therapy, N-acetylcysteine (NAC), produced no significant changes in the kidney’s function (creatinine, urea) and inflammation (IL-6, IL-10) but did produce significant changes against oxidative stress, showing lower levels of MDA, as well as a reduction of histopathologic glomerular, tubular, and interstitial damage [53].

Cadmium (Cd) is a heavy non-essential metal that is accumulated in body tissues progressively [54] and to which humans are exposed through air particles [55], occupational exposure [56] and seafood such as mollusks, crustaceans, or fish [57]. Cd can produce nephrotoxicity by many mechanisms, including DNA damage, altered gene expression, and, most importantly, oxidative damage by depleting cells’ antioxidant defenses, such as selenium, which binds to Cd to neutralize it [58]. Other proteins, e.g., metallothionein (MT), bind Cd in others to diminish its toxicity in organs such as kidneys and testis [59,60]. O_3_ therapy can diminish Cd accumulation, augment MT levels, and reduce morphologic damage, serving as an effective protective mechanism against Cd^2^⁺ renal damage [59]. It also reduces N-acetyl-β-D-glucosaminidase (NAG) [61], a lysosomal enzyme found mainly in proximal convoluted tubules, its function is the digestion of cell’s glycoconjugates [62]. The NAG increase is mediated by loss of the tubular brush border, thus liberating the enzyme into the urine [63]; such an increase is associated with pathologic processes such as Cd intoxication and malignancies of the kidney, liver, pancreas, lung, and breast, amongst many others [61,64], as well as an increased risk of requiring dialysis treatment and lethality in hospitalized patients [63]. Even when stimulating lipid peroxidation, as a result, O_3_ was also demonstrated to induce antioxidant enzymes in Cd-treated rats [65].

Some antineoplastics are proven to cause nephrotoxicity. For instance, doxorubicin, often known as Adriamycin, binds to cell membranes and inhibits nucleotide replication. However, it can be oxidized into forming reactive species like hydroxyl radicals [66]. It is demonstrated to cause severe progressive damage, fibrosis, and proteinuria [67]. O_3_ therapy, in certain doses, has proven to mediate protective effects against this morphologic damage, and arterial pressure, as well as proteinuria, have been ameliorated in rats receiving this treatment [68].

Another example is cisplatin (CDDP), an FDA (American Food and Drug Administration) approved treatment for advanced solid cancers such as that of the testis, ovary, and bladder [69]. CDDP is a molecule composed of a single platinum atom bound to chloride and ammonium; due to its small size, it filtrates freely into the glomerular barrier without tubular reabsorption [70]. It then enters tubular cells and dissociates into its toxic components, which damage DNA, membrane transporters, and mitochondrial function, thus producing oxidative stress, inflammation, and apoptosis [70,71]. O_3_ has been used as a therapy against CDDP induced damage, improving function and augmenting antioxidant defenses. Thiobarbituric acid reactive substances (TBARS, an assay used to measure lipid peroxidation; [72]), as well as NAG and morphologic damage, displayed decreased values when treated with O_3_ [73,74,75]. Protective effects, however, varied according to the administered O_3_ concentration, given that the administration of 0.36 mg/kg might be therapeutic [60] or might not [75]. On the other hand, 1.1 mg/kg always shows protective tendencies in CDDP-induced damage [73,74,75]. Higher concentrations, e.g., 1.8 mg/kg, might be protective [62]. However, due to the high formation rate of hydrogen peroxide and oxidative stress mediated by O_3_, toxic effects might be produced [73]. Very similar protective morphologic, anti-inflammatory, and antioxidant effects have been found against the damage induced by methotrexate, another cancer drug, in the kidneys, as well as the intestines and liver [76].

Radiographic contrast media (CM) is constantly used in clinical procedures which require the observation of vascular compartments. Mechanisms through which CM might cause renal dysfunction include direct oxygen-free radical damage, modified hemodynamics, and hypoxic renal medullary injury mediated by shortness of blood flow and an increase in tubular O_2_ supply. Therefore, the employment of CM produces high toxicity [77], which can be treated with O_3_. Neutrophil gelatinase-associated lipocalin (NGAL) is a damage marker observed in contrast-induced nephropathy (CIN) which augmented its expression when treated with O_3_; no further discussion was provided, although the initial oxidation by O_3_ might have produced it [78,79].

In the medical field, the use of xenobiotics as drugs to treat and diagnose diseases is an irreplaceable factor. However, during their metabolism and excretion, some might become nephrotoxic by accumulation, directing damage, the formation of free radicals, and depletion of antioxidant substances. This represents a risk for patients with neoplasia or other conditions which require constant chemical induction or those in contact with environmental components such as Cd, which is also demonstrated to cause similar renal damage. However, O_3_ is an effective treatment against this damage, at least experimentally, and thus the importance of further research in clinical environments.

**Table 2:** Ozone (O_3_) effects on chemical-induced damage models.

Damage Model	Induced Procedure	O_3_ Administration	Effects in O_3_ Treated Rats	Ref.
APAP toxicity	A 1.0 g/kg dose suspended in H_2_O, 3 mL: orally	Single i.p. 0.7 mg/kg dose at [60 mg/mL] Immediately after APAP induction	↑ SOD, GSH-Px ↓ SCr, BUN ↓ MDA ↓ Morphologic damage	[51]
APAP toxicity	A 1.0 g/kg dose suspended in H_2_O, 3 mL: gastric tube	5 daily 0.7 mg/kg doses i.p. at [60 mg/mL] Immediately after APAP induction	↑ GSH-Px, IL-10 ↓ Morphologic damage ↓ MDA ↓TNF-α	[53]
Experimental toxic adriamycin-induced glomerulonephritis	Adriamycin single 7.5 mg/kg dose through a jugular vein; 10-week evolution	After 10 weeks, daily for 15 days at 0.3 mg/kg or 0.5 mg/kg or 0.7 mg/kg, or 1.1 mg/kg	(0.3 mg/kg) ↓ Arterial pressure ↓ Proteinuria (0.5 mg/kg) ↓ Morphologic damage (0.7 and 1.1 mg/kg) No significant changes	[68]
Cd intoxication	Drinking water with Cd^2^⁺ (50 mg/L) in the form of Cadmium Acetate for 12 weeks	10 (1 daily) 1 mL i.p. doses at [40 μg/mL]	↓ Morphologic damage ↓ Glomerulonephritis ↓ NAG	[61]
Cd Intoxication	Drinking water with Cd^2^⁺ (50 mg/L) in the form of Cadmium Acetate for 12 weeks	10 (1 daily) 1 mL i.p. doses at [40 μg/mL]	↑ MT ↓ Morphologic damage	[59]
CDDP induced nephrotoxicity	Single 6 mg/kg CDDP injection	Preconditioning 15 (1 daily) doses by rectal insufflation, 9 mL at concentrations of [0.36, 0.72, 1.1, 1.8, 2.5 mg/kg]	↑ GSH, SOD, CAT, GSH-Px ↓ SCr ↓ TBARS	[73]
CDDP induced nephrotoxicity	Single 6 mg/kg CDDP injection	Postconditioning 6 (1 daily) rectal insufflations, 9 mL volume with concentrations of: 10 mg at [0.36 mg/kg] or 30 mg at [1.10 mg/kg] or 50 mg at [1.80 mg/kg]	↑ GSH, SOD, CAT, GSH-Px ↓ SCr ↓ TBARS	[75]
CDDP induced nephrotoxicity	Single 6 mg/kg CDDP injection	Daily; 5 days before and 5 days after CDDP injection. i.p.at 1.1 mg/kg	↑ CAT, SOD ↑ NAG, TGF-β1, IL-6 ↓ Morphologic damage ↓Urea, creatinine, uric acid, phosphorus, calcium, sNGAL, albumin ↓ NF-a, IL-1B,	[74]
CIN	10 mg/kg injected through the tail vein	1. 6 h before and 6 h after OR 2. For 5 days after; contrast agent introduction. O_3_ at 1 mg/kg, 95% i.p.	1. ↑ NGAL ↓Hemorrhage 2. ↑TAC, similar SCr ↓Renal tubular injury	[79]
CIN	6 mL/kg of meglumine/sodium diatrizoate through the tail vein	Five 0.7 mg/kg/d doses i.p. [70 µg/mL] For 5 days before CIN	↑ NO ↑ TAS ↓ SCr, BUN ↓ MDA ↓ Tubular necrosis	[78]

Abbreviatures: ↑: significant increase, ↓: significant decrease, APAP: acetaminophen, BUN: blood urea nitrogen, CAT: catalase, Cd: cadmium, CDDP: cisplatin, CIN: contrast-induced nephropathy, GSH: glutathione, GSH-Px: glutathione peroxidase, IL-10: interleukin 10, i.p.: intraperitoneal route, MDA: malondialdehyde, MT: metallothionine, NAG: N-acetyl-β-D-glucosaminidase, NGAL: neutrophil gelatinase-associated lipocalin, NO: nitric oxide, O_3_: ozone, SCr: serum creatinine, SOD: superoxide dismutase, TAC: total antioxidant capacity, TAS: total antioxidant system, TBARS: thiobarbituric acid reactive substances, TGF-β1: transforming growth factor β1, TNF-α: tumor necrosis factor-alpha.

### 3.3. O_3_ Therapy Protects the Kidney against CKD

CKD is a major global health issue due to its high worldwide prevalence. In 2010, an analysis showed that about 500 million adults over 20 years old suffered from this disease [80]. As its name suggests, CKD is a progressive condition in which kidney function diminishes progressively, as indicated by a lesser glomerular filtration rate (GFR) (<60 mL/min per 1.73 m^2^) or the presence of pathologic markers, such as albuminuria, hematuria, glucosuria, or other abnormalities detected by imaging, for at least three months [3]. Many factors are involved in its development, such as hypertension, pollution, glomerulonephritis, and, most importantly, type 2 diabetes mellitus [81]. In this section, the effects of O_3_ therapy against CKD will be discussed, hoping to decipher the use of new therapeutic alternatives to delay or prevent this pathology (Table 3).

Several procedures are induced in rats to simulate CKD, such as subtotal (5/6) nephrectomy, which exposes remaining renal tissue to high pressure and perfusion, eventually diminishing renal function and hence great inflammation. O_3_ can ameliorate this condition, enhancing kidney function and antioxidant status. TBARS showed higher levels, possibly due to O_3_ mediated oxidative stress [82,83]. Adenine administration also simulates CKD through its enzymatic degradation by xanthine dehydrogenase and further accumulation of the product 2,8-dihydroxyadenine (DHA) in the renal tubules, leading to inflammation and oxidative stress [84]. O_3_ ameliorated this damaging condition mainly by stimulating the expression of antioxidant enzymes and reducing inflammation [85,86].

Diabetic kidney disease (DKD) is the main cause of CKD. It is a chronic condition caused by diabetes (whether type 1 or 2) via apoptosis, formation of free radicals, advanced glycation end-products (AGES), inflammatory cytokines, and other growth molecules. [87]. Diagnosis is made essentially through diminished GFR and proteinuria in humans. Risk factors include smoking habits and high arterial pressure. The discussion of this disease becomes important since its prevalence, and therefore that of CKD, is augmenting [88]. In experimental DKD studies that use streptozotocin (STZ) as a toxic component to β-cells, O_3_ has shown beneficial anti-apoptotic and antioxidative effects in response [89,90].

**Table 3:** Ozone (O_3_) effects on chronic kidney damage models.

Damage Model	Induced Procedure	O_3_ Administration	Effects in O_3_ Treated Rats	Ref.
Adenine Induced CKD	0.75% adenine diet for 4 weeks	1.1 mg/kg at [50 μg/mL] Via rectal insufflation	↓ SCr, BUN, K, Ca ↓ Morphologic damage ↓ MCP-1, TNFα, IL-1b, IL-6 ↓TLR 4, NFkB, p65	[85]
Subtotal Nephrectomy CKD	Right nephrectomy and left subtotal ablation. 10-week evolution	1.1 mg/kg at [50 μg/mL] Via rectal insufflation Once a day for 2 weeks	↓ TNFα, IL-1β, IL-6, ↓ SCr, BUN, K, Ca ↓ Morphologic damage ↓NLRP3, NFkB, ASC, Caspase 1	[82]
Subtotal Nephrectomy CKD	Right nephrectomy and left subtotal ablation. 10-week evolution	2.5 mL at [50 μg/mL] Dose of 0.5 mg/kg Once a day for 15 days	↑ RPF, GFR ↑ SOD, CAT, GSH, TBARS ↓ Systolic arterial pressure ↓ SCr, BUN ↓ Morphologic damage	[83]
Diabetic Nephropathy	Streptozotocin induced Diabetes 6-week evolution	1.1 mg/kg [50 μg/mL] i.p.	↑ SOD, GPx, CAT ↓ BP, Hb A_1c_ % ↓ BUN, SCr, AR, MDA	[89]
Diabetic Nephropathy	Streptozotocin induced Diabetes 6-week evolution	1.1 mg/kg [50 μg/mL] once a day for 6 weeks	↓ Caspases 1, 3, 9; HIF-1α, TNF-α, Glc, morphologic damage	[90]

Abbreviatures: ↑: significant increase, ↓: significant decrease, AR: aldose reductase, ASC: apoptosis-associated speck-like protein containing a CARD, BP: blood pressure, BUN: blood urea nitrogen, Ca: calcium, CAT: catalase, CKD: chronic kidney disease, GFR: glomerular filtration rate, Glc: glucose, GPx: glutathione peroxidase, GSH: glutathione, Hb A_1c_ %: glycosylated hemoglobin, HIF-1α: hypoxia inducible factor 1α, IL: Interleukins, i.p.: intraperitoneal route, K: potassium, MDA: malondialdehyde, MCP-1: monocyte chemoattractant protein-1, NFkB: nuclear factor kappa B, NLRP3: NLR family pyrin domain containing 3, O_3_: ozone, p65: 5 kDa polypeptide, RPF: renal plasma flow, SCr: serum creatinine, SOD: superoxide dismutase, TBARS: thiobarbituric acid reactive substances, TLR 4: Toll-Like receptor 4, TNF-α: tumor necrosis factor-alpha.

CKD usually reaches an advanced terminal stage, which require therapy for replacing renal function, or dialysis, as the indicated treatment [91]. O_3_ has been shown as a coadjutant therapy to dialysis, as demonstrated by case reports in which conventional treatment did not work. For example, Biedunkiewicz and collaborators [92] described the case of a dialyzed patient with calciphylaxis-induced ulcerations who did not respond to antibiotics and surgical treatment. Ozonated autohemotherapy in concentrations of 50 µg/mL, as well as O_3_ topic administration, allowed a successful skin transplant. Authors propose that effects are mediated through O_3_ induced the synthesis of platelet-derived growth factor (PDGF), TGF-β1, and IL-8. Paolo and collaborators [93] described the case of a hemodialyzed patient who presented necrotizing fasciitis and a fatal prognosis. However, after extracorporeal blood oxygenation and ozonization (EBOO) and O_3_ topic administration, drastic wellness, including diminished hyperpyrexia and restoration of skin lesions, was reported. EBOO might be a more comfortable and practical alternative to O_3_ administration to patients over i.p. or rectal insufflation pathways, and its safety has also been proven experimentally [94]. A clinical trial in hemodialyzed patients conducted by Tylicki and collaborators [82] showed diminished GSH levels after nine weeks of O_3_ treatment, possibly caused by an augmented antioxidant system that consumes GSH. The same authors found no difference in NK cell activity after O₃ therapy, indicating it as a safe treatment in hemodialyzed patients [95]. Interestingly, another case report concluded that this therapy might cause heart failure in complex patients, such as those with CKD, diabetes, and hypertension. This association resulted from the speculation that O_3_ therapy augmented K^+^ serum levels, which, along the diminished excretion, produced sinus arrest [96]. Contrasting effects were found by Gu and collaborators [97], who treated patients suffering from chronic hepatitis with O_3_ and, while measuring kidney function, found diminished renal damage, augmented renal blood flow, and even a significant association with lesser fatalities.

**Figure 2:**
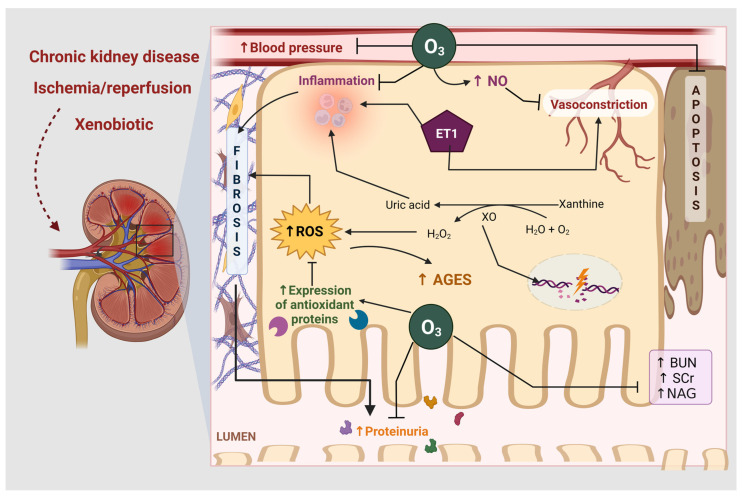
Effects of ozone therapy (O_3_) against xenobiotics, ischemia-reperfusion (IRI) and chronic kidney disease (CKD). O_3_ inhibits inflammation and ROS production by increasing the expression of antioxidant enzymes in all models. Additionally, during IRI, xanthine oxidase (XO) degrades nucleotides and forms uric acid, generating large amounts of reactive oxygen species (ROS) and inflammation. Endothelin-1 (ET-1) causes vasoconstriction and exacerbates inflammation leading to fibrosis. O_3_ therapy increases nitric oxide (NO), which inhibits vasoconstriction. While O_3_, by inhibiting ROS, causes a decrease in advanced glycation end products (AGES) and apoptosis, preventing CKD. H_2_O: water, H_2_O_2_: hydrogen peroxide, O_2_: oxygen molecule, NAG: N-acetyl-β-D-glucosaminidase. Created with Biorender.com, accessed on 10 February 2023.

To sum up, CKD is usually caused by diabetes. Both are highly prevalent, and dialysis is the standard treatment in advanced stages. O_3_ treatment is useful against these chronic diseases by reducing inflammation and oxidative stress. On top of that, O_3_ works as a coadjutant therapy for dialyzed patients to ameliorate not only kidney function, but aggravated topical microbial infections, which are common. Figure 2 shows the effects of ozone on ischemia/reperfusion, renal damage by xenobiotics, and chronic kidney disease.

### 3.4. Otherapeutic Uses of O_3_ in Kidney

Extracorporeal shock wave lithotripsy is the first-line treatment for patients with renal calculi of under 2.0 cm; therapy fragments such stones and is highly efficient. Nevertheless, adverse effects such as hematuria might be present after the procedure [98]. Experimentally, O_3_ treatment has been proven as effective against the morphological and oxidative damage caused by shock wave therapy [99]. The novel therapy, due to its antimicrobial capacity, has also ameliorated oxidative damage caused by microorganisms in kidney infection (pyelonephritis) [88] and septic shock in kidneys [100], as well as in other organs [101].

## 4. Concluding Remarks and Future Directions

Renal pathologies are currently a public global health issue, which is highly prevalent and diminishes life quality and quantity, besides being expensive for the governments and patients. After presenting this work, the conclusion that O_3_ therapy is an effective treatment against kidney injury can be stated, mainly against oxidative damage and inflammation caused by renal diseases, whether experimentally produced or in less reported clinical environments.

More research is needed to determine therapeutic regimes. The need to standardize the treatment into one most effective application comes from observing the incredible variety of doses, concentrations, and administration times amongst publications. The determination of its specific security and efficacy in humans also needs to be conducted, although adverse effects are not commonly reported when therapy is administered in the right doses; the reason why it is becoming more accepted, not only experimentally for a variety of models, but clinically as an auxiliary treatment for renal pathologies *per se*, or even other pathologies in patients on kidney support. Therefore, an exhortation to researchers to publish their O_3_ experimental results is intended; as well as an address to clinicians to publish their therapeutic O_3_ cases and, in such cases, evaluate renal markers in renal disease high-risk patients before and after the therapy, even when kidney injury is not their main therapeutic goal, since O_3_ treatment is commonly used in other clinical contexts.

## Figures and Tables

**Table 1:** Ozone (O_3_) effects on ischemic damage models.

Damage Model	Induced Procedure	O_3_ Administration	Effects in O_3_ Treated Rats	Ref.
O_3_ oxidative preconditioning Therapy				
Kidney transplantation	Right Nephrectomy and left transplant	15 (1 daily) preconditioning rectal insufflations 1 mg/kg at [50 µg/mL] to the donor rat	↑ SOD, GSH Px ↓ SCr, BUN, MDA ↓ Morphologic damage ↓ IL-6, IL-18, COX2 ↓ NF-κB, HMGB1	[45]
Kidney transplantation	Right nephrectomy and left transplant	15 (1 daily) preconditioning rectal insufflations 1 mg/kg at [50 µg/mL] to the donor rat	↑ SOD, GSH, CAT ↑ Nrf2, HO-1 ↓ SCr, BUN, MDA ↓ Morphologic damage	[46]
Right nephrectomy and left pedicle clamping	45 min ischemia 24 h reperfusion	Preconditioning therapy 15 previous rectal insufflations, 1 mg/kg at [50 µg/mL]	↓ BUN, SCr ↓ Medullar Hemorrhage ↓ TNF-α, IL-1β, IL-6, ICAM-1, ↓ MCP-1, TLR4, NF-kB	[26]
Right nephrectomy and left pedicle clamping	60 min ischemia 60 min reperfusion	Preconditioning therapy OA, 1 mL of blood added with 5 mL of O₃ [50 µg/mL] before and after IR	↑ iNOS ↑ β NADPH diaphorase ↓ BUN, SCr ↓ Medullar damage	[27]
Right nephrectomy and left pedicle clamping	45 min ischemia 24, 48, 72 h reperfusion	Preconditioning therapy 15 previous rectal insufflations, 1 mg/kg at [50 µg/mL]	↑ GSH, GSH-Px, SOD ↑ NO, iNOS, eNOS ↓ BUN, SCr ↓ Morphologic damage ↓ MDA ↓ ET-1	[28]
Right nephrectomy and left pedicle clamping	45 min ischemia 8-week reperfusion	Preconditioning therapy rectal pathway, 1 mg/kg at [50 µg/dL]	↑ SMAD-7 ↓ α- SMA, TGF-β BUN, SCr not significant	[33]
Right nephrectomy and left pedicle clamping	45 min ischemia and reperfusion	Preconditioning 15 (1 daily) doses by rectal insufflation, 1 mg/kg at [50 µg/mL]	↓ SCr, BUN, MDA ↓ Morphologic damage ↓ICAM-1, IL-1β, TNF-α, Caspase 3	[40]
Bilateral pedicle clamping	30 min ischemia and 3 h reperfusion	Preconditioning 15 (1 daily) 2.5–2.6 mL at [50 mg/mL] at a dose of 0.5 mg/kg by rectal insufflation	↑ RPF, GFR (inulin) ↑ SOD ↓ Morphologic damage	[41]
O_3_ oxidative postconditioning therapy				
Bilateral Renal Artery Occlusion	60 min ischemia 6 h reperfusion	Postconditioning therapy single 0.7 µg/kg i.p. immediately after reperfusion	↑ SOD, GSH-Px, ↓ SCr, BUN ↓ AST, Neopterin ↓ MDA, PCC, NOx ↓ Morphologic damage	[31]
Left nephrectomy and right pedicle clamping	45 min ischemia 24 h reperfusion	Postconditioning therapy 1 and 2 mg/kg; 15 (1 daily) doses after IRI at [50 μg/mL] by rectal insufflation	↑ SOD ↓ SCr, BUN, MDA ↓ Morphologic damage ↓ BAX, PARP, CREB, c-Fos	[30]
Right Nephrectomy and Left pedicle clamping	45 min ischemia 10 days reperfusion	Postconditioning therapy 10 daily rectal insufflations after IRI, a 2.5 mL volume at 0.5 mg/kg/min [50 μg/mL]	↑ SOD ↓ SCr, BUN ↓ MDA, MPO ↓ Morphologic damage ↓ α-SMA, TGF-β, p-SMAD-2	[29]
Renal vascular bundles clamping	60 min ischemia 10 days reperfusion	Postconditioning therapy Daily 10 days after IRI At 0.5 mg/kg/min via rectal insufflation	↓ Proteinuria ↑ RPF, Glomerular Filtration Rate ↓ Morphologic Damage	[32]
Bilateral Renal Artery Occlusion	60 min ischemia and 10-day reperfusion	10 (1 daily) 2.5–2.6 mL at [50 mg/mL], representing a dose of 0.5 mg/kg weight rectal insufflations	↑ CAT, SOD ↓ SCr, Fructosamine ↓ Phospholipase A2	[42]
Right nephrectomy and left pedicle clamping	45 min ischemia and 24 h reperfusion	Ischemic Preconditioning vs. O_3_ Preconditioning, 15 rectal insufflations at [50 μg/mL])	↑ NO ↑ GSH, GSP-Px, SOD ↓ BUN, SCr, MDA	[43]
Right nephrectomy and left pedicle clamping	45 min ischemia and 24 h reperfusion	Comparison Ischemic Post conditioning vs. O_3_ post conditioning, 2 mg/kg	↓ IL 1, IL 18, Caspase 1 ↓ SCr, BUN, MDA ↓ Morphologic Damage	[44]

Abbreviatures: ↑: significant increase, ↓: significant decrease, α-SMA: α-smooth muscle actin, AST: aspartate aminotransferase, BAX: bcl-2-associated X, BUN: blood urea nitrogen, CAT: catalase, COX2: cyclooxygenase 2, CREB: cAMP response element-binding, eNOS: endothelial nitric oxide synthase, ET-1: endothelin-1, FF: filtration fraction, GFR: glomerular filtration rate, GSH-Px: glutathione peroxidase, GSH: glutathione, HMGB1: high mobility group Box 1, HO-1: heme oxygenase-1, ICAM-1: intercellular adhesion molecule-1, IL-1β: interleukin- 1β, IL-6: interleukin-6, iNOS: inducible nitric oxide synthase, IRI: ischemia/reperfusion injury, MCP-1: monocyte chemoattractant protein 1, MDA: malondialdehyde, NF-kB: nuclear factor kappa B, NO: nitric oxide, O_3_: ozone, OA: ozonated autohemotherapy, PARP: polymerase 1, PCC: protein carbonyl content, RPF: renal plasma fraction, SCr: serum creatinine, SMAD-7 and -2: suppressor of mothers against decapentaplegic family members 7 and 2, SOD: superoxide dismutase, β NADPH diaphorase: β-nicotinamide adenine dinucleotide phosphate diaphorase, TGF-β: transforming growth factor β, TLR 4: Toll-Like receptor 4, TNF-α: tumor necrosis factor α.

## Data Availability

Not applicable.
